# Efficacy of a novel ultra-tapered endoscopic nasobiliary drainage tube in gallbladder drainage

**DOI:** 10.1016/j.vgie.2024.01.003

**Published:** 2024-01-25

**Authors:** Takaoki Hayakawa, Eisuke Iwasaki, Haruka Okada, Yuki Nakajima, Atsuto Kayashima, Shintaro Kawasaki, Masayasu Horibe, Takanori Kanai

**Affiliations:** 1 Division of Gastroenterology and Hepatology, Department of Internal Medicine, School of Medicine, Keio University, Tokyo, Japan; 2 Center for Diagnostic and Therapeutic Endoscopy, School of Medicine, Keio University, Tokyo, Japan

## Introduction

Endoscopic transpapillary gallbladder drainage (ETGBD) is an effective treatment for acute cholecystitis (AC) in patients who cannot undergo surgery. Mohan et al^1^ reported an 83% pooled technical ETGBD success rate. There are 2 types of ETGBD: endoscopic naso-gallbladder drainage (ENGBD) and endoscopic gallbladder stenting.^2^ ETGBD confirms bile outflow and can be removed without tube blockage using an endoscope. However, the risk of self-extraction, delicate and difficult maneuvers, and discomfort are its disadvantages.

Selective cannulation or stenting of the cystic duct can be technically difficult, especially in the presence of impacted stones and/or the tortuous nature of the cystic duct.^1^^,^^3^ Even if a guidewire is placed in the gallbladder, stent placement may fail. Unfortunately, no tubes are specifically designed for ENGBD, and regular stents with a thick tip are often used, causing unsuccessful drainage. Therefore, we developed a new ultra-tapered endoscopic nasobiliary drainage (ENBD) tube (HANACO Medical, Saitama, Japan) for ENGBD.

## Case

A 49-year-old woman had been experiencing AC frequently for about 5 years and had been treated with antibacterial agents. One year ago, she underwent ERCP to extract stones from the common bile duct. She visited the emergency department with right upper quadrant abdominal pain. The diagnosis of moderate AC was made from CT. Because she had had multiple AC episodes, emergency surgery was not indicated because of suspected severe adhesion. She was referred to our department for endoscopic conservative treatment.

## Procedure

**Figure 1:**
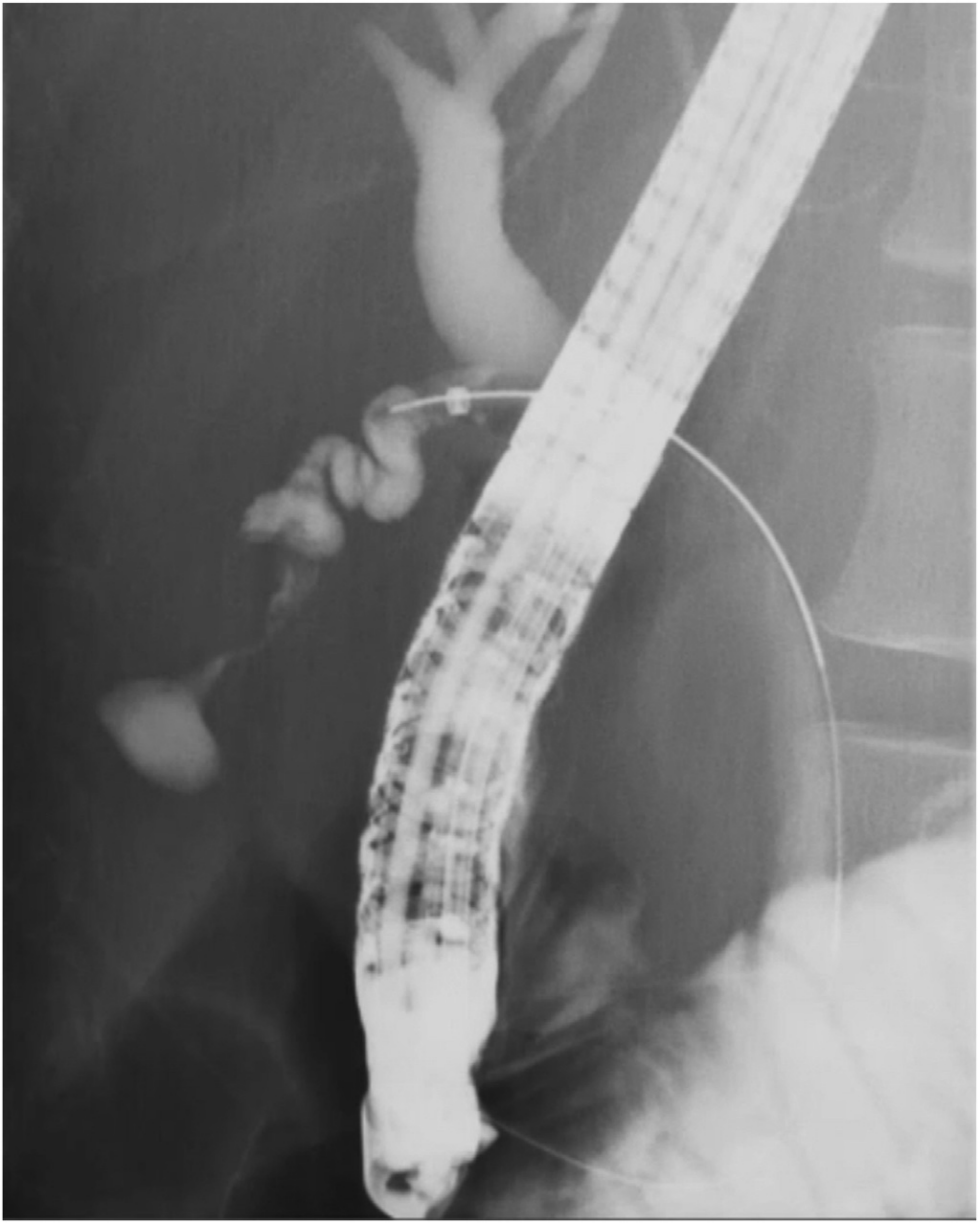
The cystic duct was highly bent, and repeated inflammation had caused stenosis, so the guidewire could not pass through. A balloon catheter (V-System single-use triple-lumen stone-extraction balloons; Olympus, Tokyo, Japan) was therefore used to inject a contrast agent in the cystic duct.

**Figure 2:**
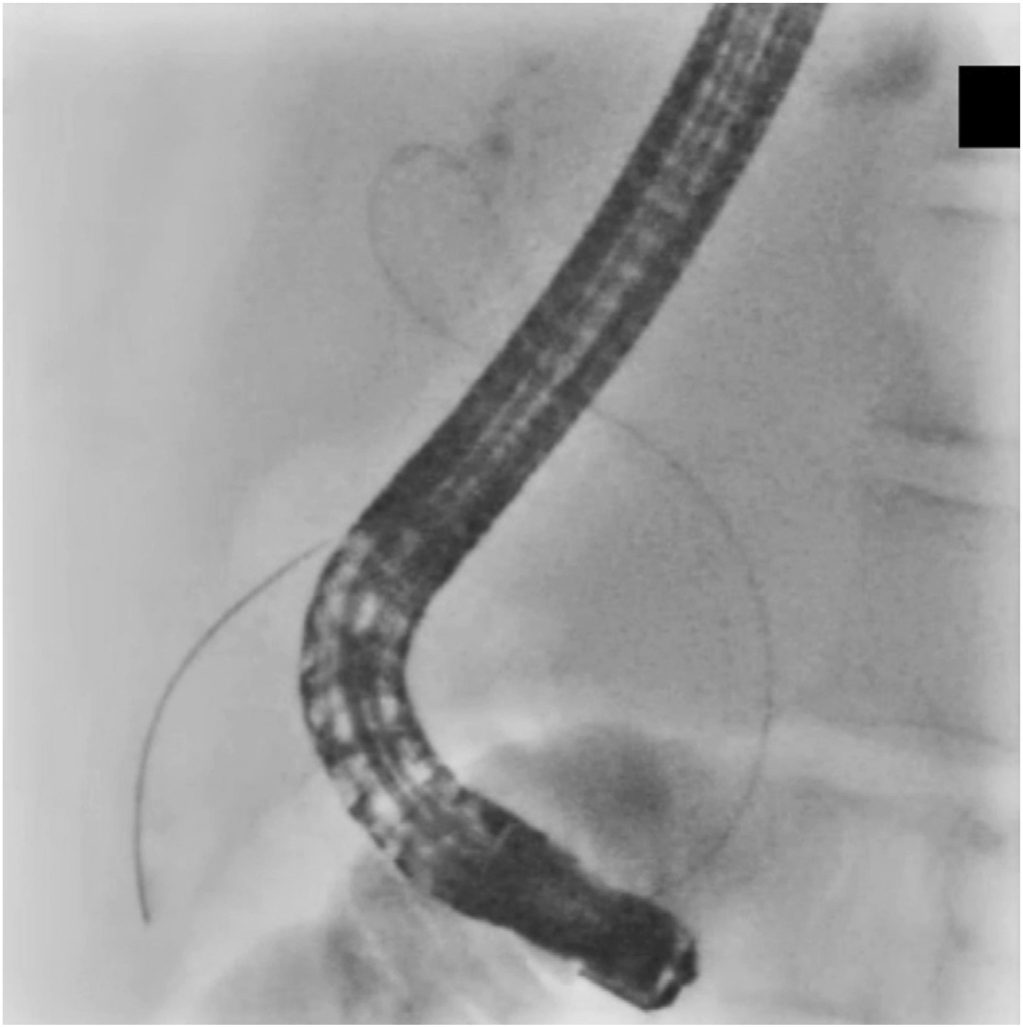
We attempted to place a conventional 5F pigtail stent, but it was difficult to pass through the cystic duct, and the stent bounced toward the liver and failed to be implanted.

**Figure 3:**
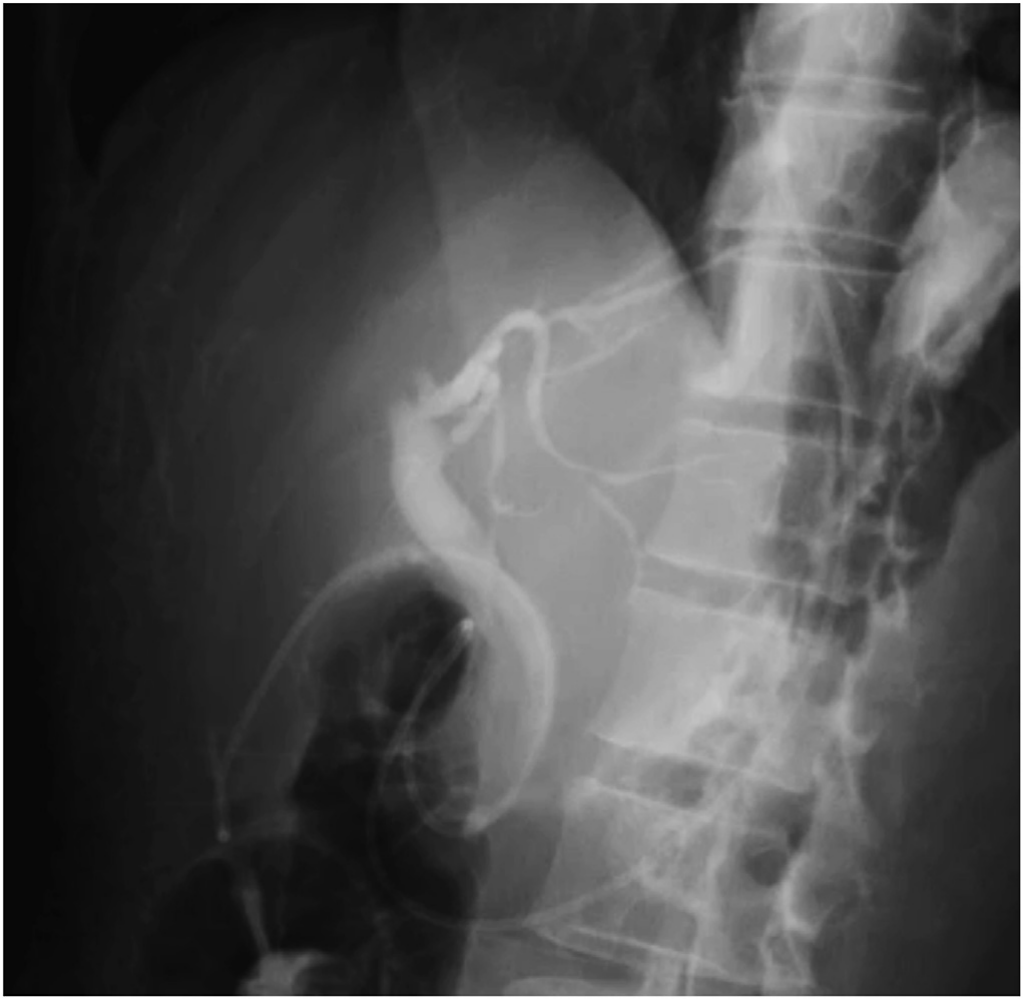
The ultra-tapered endoscopic nasobiliary drainage tube is successfully passed through the cystic duct, followed by implantation in the gallbladder.

After bile duct cannulation using a tapered catheter (MTW catheter; MTW-Endoskopie, Wesel, Germany), a conventional guidewire (Visiglide2; Olympus, Tokyo, Japan) was used successfully to hook the guidewire into the cystic duct. However, the guidewire could not pass through the cystic duct due to inflammatory benign stricture. The very narrow segment of the cystic duct was revealed by injecting a contrast agent under the balloon occlusion (V-System single-use triple-lumen stone-extraction balloons; Olympus) in the cystic duct (Fig. 1). The guidewire was changed to a hydrophilic seeking guidewire (NaviPro; Boston Scientific, Marlborough, Mass, USA), and the bottom of the gallbladder was successfully reached. Because the catheter could not follow the guidewire, we used a catheter significantly smaller in diameter (PR-110Q-1; Olympus), which successfully passed through the cystic duct. After changing the guidewire and the catheter, the gallbladder was flushed with saline solution, and the guidewire was changed to a 0.035-inch guidewire (Jagwire; Boston Scientific). We attempted to place a 5F pigtail stent, but it was difficult to pass through the cystic duct, and the stent bounced toward the hilar and failed to deploy (Fig. 2). We then tried the newly developed ultra-tapered ENBD tube over the 0.025-inch guidewire because of its “passability” and “pushability,” thereby easily and successfully performing ETGBD deployment (Fig. 3). The patient was discharged after the tube was endoscopically cut off in the stomach. Cholecystectomy was subsequently performed with the patient’s consent (Video 1, available online at www.videogie.org).

## Discussion

**Figure 4:**
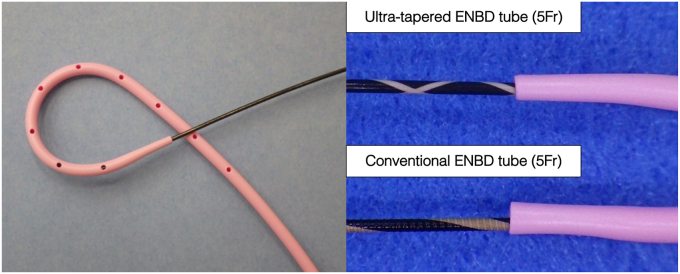
Newly developed endoscopic nasobiliary drainage (ENBD) tube (tip diameter, 3.3F; outer diameter, 5F; HANACO Medical, Saitama, Japan): an image of the tip of the newly developed ENBD and enlarged images of the tips of the conventional tubes through a 0.025-inch guidewire. The step between the ultra-tapered ENBD tube tip and the guidewire is extremely small, allowing easy passage through the cystic duct.

Although ETGBD is useful and has few adverse events,^4^ we have experienced cases with narrow and bent cystic duct resulting in procedure failure. The conventional ENBD tube has a large step when a 0.025-inch guidewire is passed through the lumen and often fails to pass through the narrow cystic duct. We developed an ultra-tapered ENBD tube (tip diameter, 3.3F; outer diameter, 5F), minimizing the step between the tip and the 0.025-inch guidewire (Fig. 4). A slightly pointed tip may cause damage to the bile and cystic ducts, but no adverse events such as bleeding have been observed in our hospital. This tube may be effective in the drainage of hilar cholangiocarcinoma with strong stenosis, and in breaking through narrow and bent cystic ducts.

We successfully performed ETGBD using a new ENBD tube in a cystic duct through which even a commonly used pigtail stent could not pass. The efficacy of the ultra-tapered ENBD tube appears promising for ETGBD. However, our findings must be confirmed in a larger number of cases.

## Conclusion

An ultra-tapered ENBD tube may be a breakthrough in the treatment of cholecystitis with a narrow cystic duct.

## Disclosure

Dr Iwasaki received grant support from Gadelius Medical. All other authors disclosed no financial relationships relevant to this publication.

## Supplementary data

Video 1 A case of endoscopic transpapillary gallbladder drainage using the ultra-tapered endoscopic nasobiliary drainage tube in a cystic duct through which even a commonly used pigtail stent could not pass.
